# Efficacy and tolerability of fixed-combination bimatoprost/timolol versus fixed-combination dorzolamide/brimonidine/timolol in patients with primary open-angle glaucoma or ocular hypertension: a multicenter, prospective, crossover study

**DOI:** 10.1186/1471-2415-14-161

**Published:** 2014-12-19

**Authors:** Alfonso García-López, José A Paczka, Jesús Jiménez-Román, Curt Hartleben

**Affiliations:** Fundación Hospital de Nuestra Señora de la Luz, Ezequiel Montes #135 C.P. 06030 Del. Cuauhtémoc, México City, D.F. México; Instituto de Oftalmología y Ciencias Visuales, CUCS, Universidad de Guadalajara, Guadalajara, México; Unidad de Diagnóstico Temprano del Glaucoma, Guadalajara, México; Asociación para Evitar la Ceguera en México (APEC) Hospital ‘Dr. Luis Sánchez Bulnes’, México City, México; Instituto de Oftalmologia Conde de Valenciana, México City, México

**Keywords:** Bimatoprost, Brimonidine, Dorzolamide, Timolol, Fixed combination, Glaucoma, Ocular hypertension

## Abstract

**Background:**

Fixed-combination ocular hypotensives have multiple advantages, but triple-therapy dorzolamide/brimonidine/timolol (dorz/brim/tim) is only available in Latin and South America, and information on its relative efficacy is limited. This study compares the efficacy and tolerability of fixed-combination bimatoprost/timolol (bim/tim) and dorz/brim/tim in Mexican patients with primary open-angle glaucoma or ocular hypertension.

**Methods:**

In this investigator-masked, crossover study, patients with unmet target intraocular pressure (IOP) on once-daily bim/tim or twice-daily dorz/brim/tim received the opposite medication for 3 months before returning to their pre-baseline medication for 3 months. IOP was evaluated before and after morning instillation at months 2, 3, 5 and 6. Primary endpoints were mean IOP change and Ocular Surface Disease Index^©^ (OSDI) score at each visit. The intent-to-treat population was the *a priori* analysis population, but due to the number of discontinuations, the per-protocol and intent-to-treat populations were used for the primary efficacy and sensitivity analyses, respectively.

**Results:**

Seventy-eight and 56 patients were included in the intent-to-treat and per-protocol populations, respectively. At month 3, statistically significant IOP reductions from baseline were observed in the bim/tim (P < 0.01) and dorz/brim/tim (*P* < 0.0001) groups, regardless of assessment time. At month 6, patients returned to bim/tim exhibited no significant IOP increase (regardless of assessment time), but patients returned to dorz/brim/tim exhibited a statistically significant IOP increase (*P* < 0.001) when assessed before instillation of study treatment. Results were similar in both intent-to-treat and per-protocol analysis populations. In the per-protocol analysis, 70% of patients on bim/tim at month 3 had an IOP <14 mm Hg, which declined to 58% (*P* = 0.0061) at month 6 (ie, after 3 months of dorz/brim/tim treatment). In patients receiving dorz/brim/tim at month 3, 38% had an IOP <14 mm Hg, which remained comparable after return to bim/tim. OSDI scores and incidence of adverse events were similar in both groups.

**Conclusions:**

In this first direct comparison of the efficacy of dorz/brim/tim and bim/tim, patients switched from dorz/brim/tim to bim/tim demonstrated improved/lower IOP; when returned to dorz/brim/tim, IOP increased to levels seen at study initiation, suggesting that once-daily bim/tim may have greater IOP-lowering efficacy. Both bim/tim and dorz/brim/tim were well tolerated with minimal ocular surface damage.

**Trial registration:**

ClinicalTrials.gov: NCT01737853 (registered October 9, 2012)

## Background

Glaucoma is a leading cause of blindness worldwide, with prevalence varying across different populations [1]. Latin Americans, especially those of Mexican origin, are prone to develop primary open-angle glaucoma (POAG), compared with Whites [2–4], and an increase in intraocular pressure (IOP) raises the risk that individuals with ocular hypertension (OHT) will develop POAG [5, 6], and that those with glaucoma will experience further progression [7, 8]. Consequently, treatment of glaucoma and OHT focuses mainly on lowering IOP [9, 10].

Options for medical therapy have expanded over the last 2 decades; beta-adrenoceptor antagonist and prostaglandin analog/prostamide monotherapies are used as first-line treatment [11] while alpha-2 adrenoceptor agonists, parasympathomimetics, and topical carbonic anhydrase inhibitors are used as second-line options [9, 10]. The introduction of these new classes of IOP-lowering drugs has contributed to challenges for prescribers, as a suitable agent depends not only on its IOP-lowering capacity, but also on its tolerability and convenience of use [12].

Monotherapy is the recommended initial approach for glaucoma treatment [13–15], but combinations of agents are often necessary [16–18]. A recently published systematic review and meta-analysis of 5 studies comparing fixed and unfixed combinations of timolol and a prostaglandin analog found that fixed combinations were less effective in reducing IOP than unfixed combinations [19]. However, statistical heterogeneity analysis suggested that this observed effect was likely due to differences in study design and conduct, and not chance alone (I^2^ = 52%). Fixed-combination hypotensive therapies do have advantages of limiting preservative-related adverse effects and producing lower rates of hyperemia [19], and have the potential to increase adherence and reduce costs [12, 18, 20].

The majority of non-prostaglandin analog-based fixed combinations available are dual, timolol-containing therapies. An exception is the triple-combination of dorzolamide 2%, brimonidine 0.2%, and timolol maleate 0.5% (dorz/brim/tim; Krytantek®; Laboratorios Sophia, Guadalajara, Mexico), which is only available in Latin and South American countries. Reports on the clinical properties of this 3-drug combination are limited [21, 22]. In contrast, the efficacy and safety of the commonly used fixed combination of bimatoprost 0.03% and timolol maleate 0.5% (bim/tim; Ganfort®; Ganforti®; Allergan, Inc., Irvine, CA, USA) has been studied extensively [23–32].

The objective of this clinical trial was to compare the efficacy and tolerability of once-daily bim/tim with twice-daily dorz/brim/tim over a 6-month period, using a crossover study design in patients with POAG or OHT who had not reached target IOP.

## Methods

**Figure 1:**
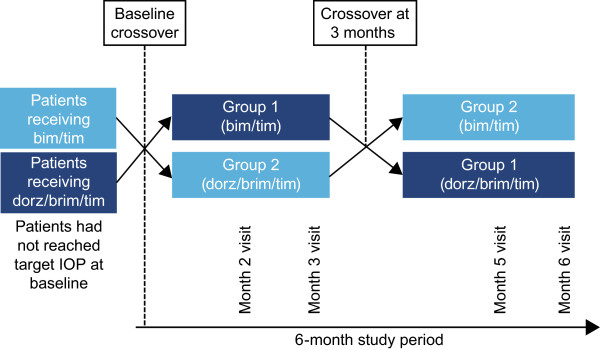
**Study design.** No assessments were performed at months 1 and 4. *Bim/tim*, bimatoprost 0.03% and timolol maleate 0.5%; *dorz/brim/tim*, dorzolamide 2%, brimonidine 0.2%, and timolol maleate 0.5%; *IOP*, intraocular pressure.

This phase 4, 6-month, prospective, multicenter, investigator-masked, crossover study (ClinicalTrials.gov Identifier: NCT01737853 [registered Oct. 9, 2012]) comprised 2 treatment periods of 3 months each (Figure 1). Patients were enrolled between February 2011 and June 2012 at 4 centers in Mexico, after the study protocol was approved by the Hospital Fundación Nuestra Señora de la Luz Ethics Committee for all study sites. The study was conducted in accordance with Good Clinical Practice guidelines, as well as all applicable local laws. Patients (or their legal guardian) signed an informed consent prior to study initiation.

### Study participants

Patients attended an initial screening visit for assessment of inclusion and exclusion criteria. The study recruited patients >17 years of age with a diagnosis of mild to moderate POAG or OHT who had failed to reach target IOP despite receiving bim/tim or dorz/brim/tim for at least 1 month. Patients were required to have an IOP ≥18 and ≤36 mm Hg, as well as a best-corrected visual acuity of at least 20/80 in both eyes. Women of reproductive age were required to use a reliable method of contraception during the study period. Exclusion criteria were: any ocular disorder other than mild to moderate loss of lens transparency, glaucoma or OHT; significant visual field loss in the previous year; uncontrolled systemic disease; active ocular disease, or intraocular surgery within the past 3 months; use of other medications with a potential substantial effect on IOP; allergy or other contraindications to any components of the study product; and severe glaucoma according to the Hodapp-Parrish-Anderson criteria [33].

### Treatment and assessments

Eligible patients who had received dorz/brim/tim for ≥1 month prior to the baseline visit (group 1) were assigned to receive bim/tim for 3 months, and those who had received bim/tim for ≥1 month prior to the baseline visit (group 2) were assigned to receive dorz/brim/tim for 3 months (Figure 1). At the 3-month visit, patients were reassigned to the study medication they had been receiving before the baseline crossover for 3 additional months. Bim/tim was administered once daily at 8 am and dorz/brim/tim was administered twice daily at 8 am and 8 pm. The investigators were masked to the instillation schedule.

Bottles of study medication were dispensed to the patient in a closed, labeled box by an office assistant, and were returned in the same box to the assigned office assistant, but never to the principal investigator or sub-investigator in order to preserve the single-masked nature of the study. Study medication bottles were dispensed at the baseline visit and monthly thereafter. Patients received verbal instructions, written reminders, and periodic phone calls to promote adherence to medication.

Assessment visits were scheduled at baseline, months 2 and 3 during the first treatment period, and months 5 and 6 during the second treatment period (Figure 1). There were no assessments at months 1 and 4 (considered the run-in phases after each crossover). At baseline and study end, IOP, visual acuity, refraction and visual field (Humphrey 24–2) were evaluated, and central ultrasound pachymetry, gonioscopy, and dilated funduscopy were performed. IOP assessments were performed (using a Goldmann applanation tonometer) at 8 am and 10 am (ie, before and after morning instillation of the study drug) and recorded at each visit, along with Ocular Surface Disease Index^©^ (OSDI) [34], slit-lamp biomicroscopy, blood pressure, pulse, and adverse events. Since IOP was a main study endpoint, particular care was taken to obtain unbiased measurements using a 2-person method in which one adjusts the dial in a masked fashion and the second reads and records the value. IOP was measured twice consecutively; if the difference between measurements was >2 mm Hg, a third measurement was taken, and the mean of all 3 measurements was recorded.

### Data analysis and sample size calculation

The primary endpoints were mean IOP reduction (at 8 am and 10 am) and OSDI scores at each visit. The intent-to-treat (ITT) population was the *a priori* analysis population, but due to the considerable number of discontinuations, the per-protocol (PP) population was used for the primary efficacy and OSDI analyses, and the ITT population was used for sensitivity analyses. For patients with a bilateral condition, data for a randomly chosen eye were included in the analyses.

Analyses of mean IOP and differences in IOP reduction between treatment arms were performed using a repeated analysis of variance. Intra-group comparisons of values at baseline and subsequent time-points were conducted using a 2-tailed, paired Student *t* test. A Bonferroni post hoc correction was used to adjust the *P* value for individual time-points. The percentage of patients with IOP control at baseline and months 3 and 6 (10 am assessment) was calculated for each of the treatment arms, based on IOP level (<14, <18, and <21 mm Hg). A chi-square distribution test was used to determine the statistical significance of the change in percentage in the response analysis. The Student *t* test and a nonparametric chi-square test were used to analyze between-group differences in OSDI scores and adverse events, respectively, at each time-point in the ITT population.

Enrollment of 90 patients was planned (45 per treatment arm) to ensure approximately 80 evaluable patients (40 per treatment arm). The sample size calculation took into account the following assumptions: a standardized effect size of 0.60, a 2-sided α value of 0.01, and a β value of 0.10.

## Results

Ninety-two patients were screened and 85 enrolled; 7 patients did not meet the eligibility criteria at baseline. Of the 78 patients who received 1 dose of study medication (ITT population), 22 discontinued early. Reasons recorded were nonadherence to therapy (n = 10), unwillingness to participate (n = 6), or the following adverse events: 3 patients with severe hyperemia or sensation of foreign body while receiving bim/tim, 2 patients with severe hyperemia or reduced visual field (caused by treatment-unrelated cerebrovascular disease; the patient recovered total visual capacity after 48 hours) while receiving dorz/brim/tim; 1 patient with an unspecified adverse event. Fifty-six patients comprised the PP population. Patient demographics and characteristics are presented in Table 1. Mean age ± standard deviation was 66 ± 10 years (range: 46–93), the majority of patients were female, and 62% had a diagnosis of POAG.

**Table 1:** **Patients demographics and characteristics at baseline**

Characteristics	ITT population (N = 78)	PP population (N = 56)
Mean age, years (SD)	66 (10)	66 (10)
Female, n (%)	64 (82)	46 (82)
Race		
Hispanic, n (%)	78 (100)	56 (100)
Baseline IOP, mm Hg (SD)	19.5 (2.2)	19.5 (2.1)
BCVA, letters (SD)	0.3 (0.3)	0.2 (0.3)
Corneal thickness, μm	545 ± 36	540 ± 36

BCVA, best-corrected visual acuity; ITT, intent-to-treat; OHT, ocular hypertension; POAG, primary open-angle glaucoma; PP, per-protocol; SD, standard deviation.

In the PP population, patients who received bim/tim for the first 3 months (group 1) had a mean baseline IOP of 19.5 ± 2.1 mm Hg at 8 am and 18.3 ± 2.6 mm Hg at 10 am. At the 3-month visit, mean IOP was reduced to 14.6 ± 3.8 mm Hg at 8 am and 13.6 mm Hg ± 2.8 at 10 am (Table 2). Patients who received dorz/brim/tim for the first 3 months (group 2) had a mean baseline IOP of 20.2 ± 2.5 and 19.4 ± 1.9 mm Hg at 8 am and 10 am, respectively. At the 3-month visit, mean IOP was reduced to 16.9 ± 3.7 and 15.7 ± 2.9 mm Hg at 8 am and 10 am, respectively (Table 2). Statistically significant IOP reductions from baseline were observed in both groups at the month 3 visit, regardless of the assessment time (Table 3).

**Table 2:** **Comparison of the mean intraocular pressure at each assessment visit in the per-protocol and intent-to-treat populations**

Per-protocol population								
Treatment group/time-point (baseline)	Mean IOP ± SD (mm Hg)			n	Treatment group/time-point (crossover)	Mean IOP ± SD (mm Hg)		n
	Baseline	Month 2	Month 3			Month 5	Month 6	
Bim/tim 8 am	19.5 ± 2.1	15.9 ± 3.6	14.6 ± 3.8	30	Dorz/brim/tim 8 am	16.6 ± 4.0	17.6 ± 3.4	30
Bim/tim 10 am	18.3 ± 2.6	14.2 ± 3.1	13.6 ± 2.8		Dorz/brim/tim 10 am	13.3 ± 3.2	13.8 ± 3.1	
Dorz/brim/tim 8 am	20.2 ± 2.5	17.1 ± 3.4	16.9 ± 3.7	26	Bim/tim 8 am	17.0 ± 3.3	16.0 ± 2.9	26
Dorz/brim/tim 10 am	19.4 ± 1.9	15.8 ± 2.9	15.7 ± 2.9		Bim/tim 10 am	16.0 ± 2.9	15.6 ± 2.3	
**Intent-to-treat population**								
**Treatment group/time-point (baseline)**	**Baseline**	**Month 2**	**Month 3**	**n**	**Treatment group/time-point (crossover)**	**Month 5**	**Month 6**	**n**
Bim/tim 8 am	19.5 ± 2.2	16.1 ± 3.4	14.8 ± 3.5	41 (BL)–35 (M3)	Dorz/brim/tim 8 am	16.5 ± 3.8	17.7 ± 3.4	35 (M3)–26 (M6)
Bim/tim 10 am	18.1 ± 2.7	14.5 ± 2.3	13.7 ± 2.3		Dorz/brim/tim 10 am	13.3 ± 3.0	14.1 ± 3.3	
Dorz/brim/tim 8 am	19.8 ± 2.3	16.8 ± 3.7	16.7 ± 3.7	37 (BL)–28 (M3)	Bim/tim 8 am	16.7 ± 3.3	16.6 ± 2.9	28 (M3)–32 (M6)
Dorz/brim/tim 10 am	18.6 ± 2.9	15.3 ± 2.6	15.6 ± 2.9		Bim/tim 10 am	15.7 ± 3.1	15.6 ± 2.3	

*Bim/tim*, bimatoprost 0.03% and timolol maleate 0.5%; BL, baseline; *dorz/brim/tim*, dorzolamide 2%, brimonidine 0.2%, and timolol maleate 0.5%; M3, month 3; M6, month 6, SD, standard deviation.

**Table 3:** **Differences in mean intraocular pressure with each treatment**

Per-protocol population							
Treatment group/time-point (baseline)	Baseline-month 3			Treatment group/time-point (crossover)	Month 3-month 6		
	mm Hg ± SD	***P***value*	n		mm Hg ± SD	***P***value*	n
Bim/tim 8 am	-4.9 ± 1.7	0.0073	30	Dorz/brim/tim 8 am	+3.0 ± 0.4	0.0006	30
Bim/tim 10 am	-4.7 ± 0.2	0.0045		Dorz/brim/tim 10 am	+0.2 ± 0.3	0.2710	
Dorz/brim/tim 8 am	-3.3 ± 1.2	<0.0001	26	Bim/tim 8 am	-0.9 ± 0.8	0.0770	26
Dorz/brim/tim 10 am	-3.0 ± 1.0	<0.0001		Bim/tim 10 am	0.0 ± 0.6	0.9463	
**Intent-to-treat population**							
**Treatment group/time-point (baseline)**	**Baseline-month 3**			**Treatment group/time-point (crossover)**	**Month 3-month 6**		
	**mm Hg ± SD**	***P*** value*	**n**		**mm Hg ± SD**	***P*** value*	**n**
Bim/tim 8 am	-4.7 ± 1.3	<0.0001	41 (BL)–35 (M3)	Dorz/brim/tim 8 am	+2.9 ± 2.9	0.0006	35 (M3)–26 (M6)
Bim/tim 10 am	-4.4 ± 0.4	<0.0001		Dorz/brim/tim 10 am	+0.3 ± 0.3	0.5940	
Dorz/brim/tim 8 am	-3.1 ± 1.3	<0.0001	37 (BL)–28 (M3)	Bim/tim 8 am	-0.1 ± 0.8	0.9286	28 (M3)–32 (M6)
Dorz/brim/tim 10 am	-3.0 ± 0.0	<0.0001		Bim/tim 10 am	0.0 ± 0.6	0.9463	

*Student *t* test.

*Bim/tim*, bimatoprost 0.03% and timolol maleate 0.5%; BL, baseline; *dorz/brim/tim*, dorzolamide 2%, brimonidine 0.2%, and timolol maleate 0.5%; M3, month 3; M6, month 6; SD, standard deviation.

**Figure 2:**
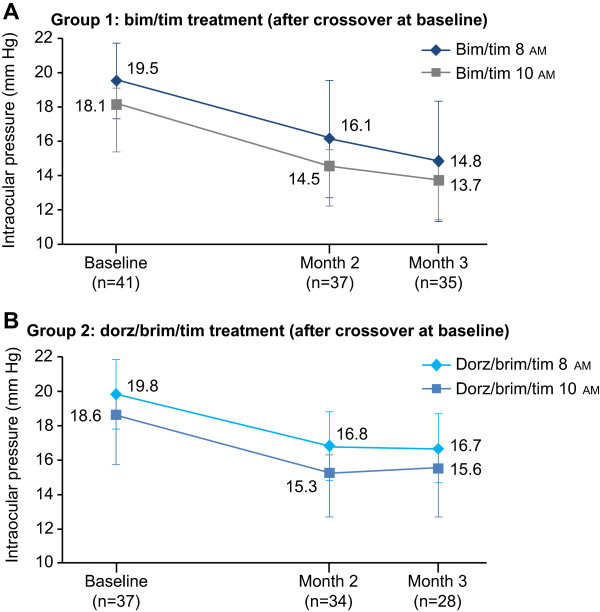
**Mean IOP at baseline, month 2, and month 3 following the baseline crossover (ITT population). A**. Mean IOP ± SD in Group 1. **B**. Mean IOP ± SD in Group 2. *Bim/tim*, bimatoprost 0.03% and timolol maleate 0.5%; *dorz/brim/tim*, dorzolamide 2%, brimonidine 0.2%, and timolol maleate 0.5%; *IOP*, intraocular pressure; *ITT*, intent-to-treat; SD, standard deviation.

**Figure 3:**
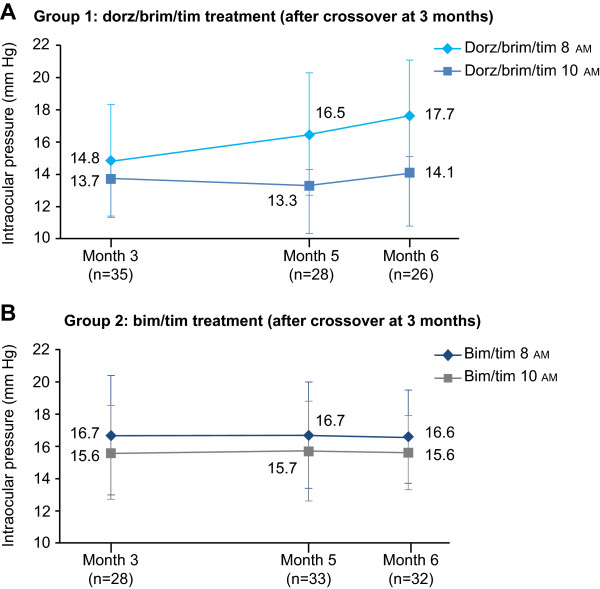
**Mean IOP at months 3, 5 and 6 following the 3-month crossover (ITT population). A**. Mean IOP ± SD in Group 1. **B**. Mean IOP ± SD in Group 2. *Bim/tim*, bimatoprost 0.03% and timolol maleate 0.5%; *dorz/brim/tim*, dorzolamide 2%, brimonidine 0.2%, and timolol maleate 0.5%; *IOP*, intraocular pressure; *ITT*, intent-to-treat; SD, standard deviation.

When patients assigned to bim/tim at baseline were switched to dorz/brim/tim at 3 months (group 1), mean IOP at 8 am increased from 14.6 ± 3.8 mm Hg to 17.6 ± 3.4 mm Hg at the 6-month visit, but remained stable at 10 am (13.6 ± 2.8 to 13.8 ± 3.1 mm Hg, respectively). When patients assigned to dorz/brim/tim at baseline were switched to bim/tim at 3 months (group 2), IOP was relatively stable up to month 6 (Tables 2 and 3). Importantly, similar results were observed in the ITT population (Figures 2A-B, 3A-B, and Tables 2 and 3).

When the change in IOP observed during the second crossover period was analyzed by visit and measurement time, mean IOP significantly increased from months 3 to 5 in all but the dorz/brim/tim group at 10 am; from months 5 to 6, there was a statistically significant decrease in IOP in the bim/tim-treated group at both 8 and 10 AM (Table 4). However, a similar analysis in the ITT population did not reveal any statistically significant changes in IOP between visits.

**Table 4:** **Differences in mean intraocular pressure between visits during the second crossover period**

Per-protocol population					
Treatment group/time-point	Month 3-month 5		Month 5-month 6		n
	mm Hg ± SD	***P***value*	mm Hg ± SD	***P***value*	
Dorz/brim/tim 8 am	+2.0 ± 0.2	0.004	+1.0 ± 0.5	0.035	30
Dorz/brim/tim 10 am	-0.3 ± 0.4	0.036	+0.5 ± 0.1	0.031	
Bim/tim 8 am	+0.1 ± 0.4	0.007	-1.0 ± 0.4	0.003	26
Bim/tim 10 am	+0.3 ± 0.1	0.031	-0.4 ± 0.5	0.015	

*Student *t* test.

*Bim/tim*, bimatoprost 0.03% and timolol maleate 0.5%; *dorz/brim/tim*, dorzolamide 2%, brimonidine 0.2%, and timolol maleate 0.5%; SD, standard deviation.

From baseline to month 3, there were statistically significant increases in the percentage of patients with IOP <14 mm Hg and <18 mm Hg among those receiving bim/tim (group 1) and dorz/brim/tim (group 2) (Table 5). Notably, similar responses were observed in the ITT population. Between months 3 and 6, there was a statistically significant decline in the percentage of patients with IOP <14 mm Hg and <18 mm Hg after switching from bim/tim to dorz/brim/tim (*P* ≤ 0.0061) in both the PP and ITT populations. In contrast, there was no such decline after patients switched from dorz/brim/tim to bim/tim (Table 5). During the first treatment period, the percentage of patients with IOP <14 mm Hg while receiving bim/tim was almost twice that of patients receiving dorz/brim/tim. During the second treatment period, this difference in response was somewhat maintained, favoring patients receiving dorz/brim/tim (Table 5). Overall, the response to bim/tim and dorz/brim/tim was significantly different from baseline to month 3 and from months 3 to 6 (*P* ≤ 0.014 and *P* ≤ 0.008, respectively) in both the PP and ITT analyses.

**Figure 4:**
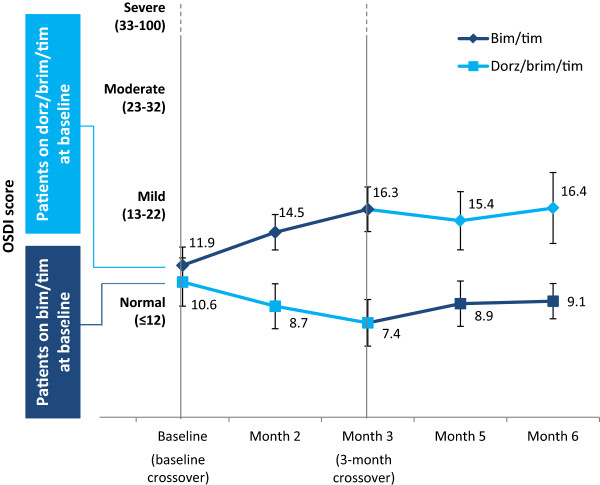
**Mean OSDI scores over the course of the study (n = 78).** The change in OSDI score (± SE) over time was not statistically significant in either group (P > 0.1). *Bim/tim*, bimatoprost 0.03% and timolol maleate 0.5%; *dorz/brim/tim*, dorzolamide 2%, brimonidine 0.2%, and timolol maleate 0.5%; *OSDI*, Ocular Surface Disease Index; SE, standard error.

During the course of the study, OSDI scores were similar in both treatment arms (Figure 4). Of note, although still in the normal range, the mean baseline score in patients receiving dorz/brim/tim prior to the baseline crossover was higher than in those receiving bim/tim and tended to increase over time.

**Table 5:** **Response to treatment by level of IOP**

Per-protocol population								
IOP level (mm Hg, 10 **am**measurement)*	Treatment group	Percentage of patients		IOP change Baseline–month 3 ***P***value ^†^	Treatment group	Percentage of patients	IOP change Months 3–6 ***P***value ^†^	n
		Baseline	Month 3			Month 6		
<14	Bim/tim (group 1)	3	70	<0.00001	Dorz/brim/tim (group 1)	58	0.0061	30
<18		31	100			93		
<21		95	100			100		
<14	Dorz/brim/tim (group 2)	0	38	<0.0001	Bim/tim (group 2)	33	0.1778	26
<18		30	93			87		
<21		85	98			96		

*The presence of patients in the <14 and <18 mm Hg categories at baseline is due to the fact that baseline IOP was assessed at 8 AM (ie, before instillation) whereas response to treatment was based on IOP measurements taken at 10 AM (ie, 2 hours post-instillation).

^**†**^2-tailed, chi-square distribution.

*Bim/tim*, bimatoprost 0.03% and timolol maleate 0.5%; *dorz/brim/tim*, dorzolamide 2%, brimonidine 0.2%, and timolol maleate 0.5%; *IOP*: Intraocular pressure.

Few adverse events were reported during the study, with severe hyperemia being the most common and the only one that was treatment-related (Table 6). Otherwise, cup/disk ratio and visual field were not statistically different between treatment groups. Cup/disk ratios were 0.63 vs 0.65 at baseline, and 0.63 vs 0.64 at the final visit for groups 1 (*P* = 0.46) and 2 (*P* = 0.75), respectively. Similarly, pattern standard deviation was 4.03 vs 4.06 at baseline, and 4.44 vs 4.38 at the final visit for groups 1 (*P* = 0.96) and 2 (*P* = 0.91), respectively.

**Table 6:** **Adverse events reported during the study (N = 78)**

Adverse event	Cases	Withdrawals	Treatment at the time of withdrawal*
Vitreous hemorrhage	1	0	
Pruritus	1	0	
Severe hyperemia	3	3	bim/tim (2), dorz/brim/tim (1)
Sensation of foreign body and conjunctival edema	1	1	bim/tim
Severe eyelid edema	1	0	
Reduced visual field	1	1	dorz/brim/tim
Allergy	1	0	
Significantly reduced vision	1	0	
Blurred vision	1	0	
Not specified	1	1	dorz/brim/tim
Total	12	6	

*Refers to the study medication taken at the time of withdrawal, but does not indicate that the adverse event was treatment-related.

Nonparametric chi-square test, *P* = 0.202.

*Bim/tim*, bimatoprost 0.03% and timolol maleate 0.5%; *dorz/brim/tim*, dorzolamide 2%, brimonidine 0.2%, and timolol maleate 0.5%.

## Discussion

In patients with POAG or OHT and inadequate IOP control on once-daily bim/tim or twice-daily dorz/brim/tim, significant IOP reductions from baseline were produced over a 3-month period when transitioned to the opposite therapy. However, bim/tim demonstrated better IOP reduction at peak (10 am) and trough (8 am) than dorz/brim/tim. At the end of the second treatment period (ie, month 6), when treatment was returned to the combination used prior to the baseline crossover, nonsignificant IOP reductions from month-3 were seen at both peak and trough in patients receiving bim/tim. In contrast, patients receiving dorz/brim/tim experienced an IOP increase at trough between both months 3 and 5, and 5 and 6, most likely due to the pharmacokinetics of dorz/brim/tim itself. During the first treatment period, a significant percentage of patients achieved an IOP response <14 mm Hg in both treatment groups, compared with baseline, but the percentage was almost twice as high in the bim/tim group. In addition, the level of IOP lowering was maintained in patients switched from dorz/brim/tim to bim/tim during the second treatment period. Importantly, findings of the primary efficacy and OSDI analyses were confirmed in a sensitivity analysis using the ITT population.

Clinicians have become increasingly interested in the effects of topical treatments on the condition of the ocular surface in patients with glaucoma and OHT. Chronic use of eye drops, particularly multidose products that contain preservatives, has been associated with damage to the ocular surface owing to the inherent toxicity of some preservatives and drugs [35–38]. As demonstrated by the low OSDI scores (the gold standard measure of ocular surface damage [34]), both bim/tim and dorz/brim/tim had minimal, if any, detrimental effects on the ocular surface. OSDI scores of patients who entered the study on dorz/brim/tim tended to increase slightly over time, especially during the first treatment period. The underlying reason is unclear as the OSDI score remained relatively stable after a similar switch from dorz/brim/tim to bim/tim in patients receiving bim/tim prior to the baseline crossover.

Treatment of glaucoma or OHT aims to lower IOP and preserve visual function. The benefits of lowering IOP in delaying or preventing disease progression are well documented [9, 39–41]. A Canadian observational study has shown that in patients with progressive disease, a 20% reduction in median IOP was associated with a 69% reduction in the median rate of visual field decline [42]. A large number of IOP-lowering medications are available, and the choice of a suitable product depends not only on its IOP-lowering capacity, but also on its tolerability and convenience of use. The advantages of fixed-combination treatments such as bim/tim and dorz/brim/tim over unfixed combinations (ie, multiple single agents used concomitantly) are well known [43, 44], but information on the relative efficacy of dorz/brim/tim is limited. The findings of our study suggest that bim/tim may offer benefits over dorz/brim/tim in terms of IOP lowering. The once-daily administration of bim/tim may also be more convenient for patients and thus lead to improved adherence to therapy [45], although neither convenience nor adherence was evaluated in this study. In future studies, the increase in IOP observed with dorz/brim/tim after the second crossover is a phenomenon that should be investigated. Moreover, the longer-term effects of dorz/brim/tim on IOP should be evaluated to determine the advantages and disadvantages of this combination in the treatment of patients with chronic open-angle glaucoma.

Since low diastolic blood pressure and diastolic ocular perfusion pressure have also been reported as risk factors for the development and progression of POAG [3, 46–49], the effect of topical IOP-lowering therapies on these variables has been investigated. Two randomized clinical studies of patients with treatment-naive POAG and no history of cardiovascular disease found that timolol and brimonidine monotherapies, and fixed-combination timolol/dorzolamide significantly decreased diastolic blood pressure over 24 hours; dorzolamide or latanoprost alone had either no or opposite effects on blood pressure, and increased calculated diastolic ocular perfusion pressure [50, 51]. A later, similarly designed study reported that timolol had minimal effects on blood pressure and calculated ocular perfusion pressure [52], suggesting that additional, long-term confirmatory research is needed. Nevertheless, these studies appear to support a better risk-to-benefit ratio for bim/tim than for dorz/brim/tim in the present study.

A particular strength of our study is the use of a crossover design, which minimizes the impact of confounding covariates while requiring fewer patients to achieve the same precision as a parallel-group trial [53, 54]. Nevertheless, crossover trials have some recognized disadvantages, such as the potential for carryover effects between treatments. In our study, however, the potential for carryover effects was addressed by including a wash-in period and initiating assessment of outcome variables 2 months after the first crossover (month 3) and second crossover (month 5) of study medications. Another potential weakness relates to the considerable number of discontinuations recorded over the course of the study. Although we cannot exclude the possibility that patients who discontinued because of nonadherence or unwillingness to participate did so after experiencing adverse events, our calculations indicated that a 95% confidence level would have required a total of 74 patients, whereas 39 patients would suffice to attain a 90% confidence level. We can thus conclude that the number of discontinuations did not limit the statistical significance of our findings or the study conclusions.

## Conclusions

Topical combination therapies are often necessary to prevent onset or progression of glaucoma, and fixed-combinations are advantageous over concomitant single agents. To the best of our knowledge, this is the first, direct comparison of fixed-combination dorz/brim/tim and bim/tim published. In patients with POAG or OHT and inadequate IOP control, both once-daily bim/tim and twice-daily dorz/brim/tim reduced IOP, although once-daily bim/tim appeared to offer greater IOP-lowering efficacy. Bim/tim and dorz/brim/tim were well tolerated and had minimal (if any) detrimental effects on the ocular surface. Further studies are needed to compare the longer-term effects on ocular surface and adherence, especially given the twice-daily dosing regimen of dorz/brim/tim.

## Abbreviations

Bim/tim: bimatoprost 0.03% and timolol maleate 0.5%
dorz/brim/tim: dorzolamide 2%, brimonidine 0.2%, and timolol maleate 0.5%
IOP: intraocular pressure
OHT: ocular hypertension
OSDI: Ocular Surface Disease Index
POAG: primary open-angle glaucoma.
